# 4,4-Dimethyl-2-phenyl-4,5-di­hydro­pyrrolo­[2,3,4-*kl*]acridin-1(2*H*)-one

**DOI:** 10.1107/S241431462500361X

**Published:** 2025-04-24

**Authors:** Izarul Islam, Pijush Kanti Roy, Ennio Zangrando, Pran Gopal Karmaker, Harendra Nath Roy

**Affiliations:** a Department of Chemistry, University of Rajshahi, Rajshahi-6205, Bangladesh; b Department of Chemistry, Faculty of Science, Mawlana Bhashani Science and Technology University, Tangail-1902, Bangladesh; c Department of Chemical and Pharmaceutical Sciences, University of Trieste, Italy; d Chemical Synthesis and Pollution Control Key Laboratory of Sichuan Province College of Chemistry and Chemical Engineering China West Normal University,Nanchong 637002 People’s Republic of China; University of Aberdeen, United Kingdom

**Keywords:** crystal structure, pyrrolo­acridine, fused heterocyclic compound

## Abstract

In the title compound, C_22_H_18_N_2_O, the pendant phenyl ring is twisted by 43.85 (1)° with respect to the acridine moiety, which has almost coplanar atoms apart from the *sp*^3^ carbon atoms. The extended structure features aromatic π–π stacking with a centroid-to-centroid distance of 3.489 (2) Å and weak C—H⋯O hydrogen bonds.

## Abstract 2

In the title compound, the pendant phenyl ring is twisted by 43.85 (1)° with respect to the acridine moiety. The extended structure features aromatic π–π stacking with a centroid-to-centroid distance of 3.489 (2) Å and weak C—H⋯O hydrogen bonds.

## Graphical abstract

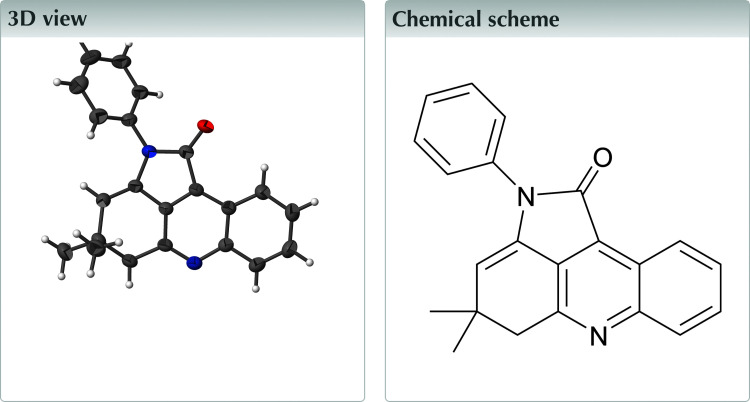

## Structure description

Pyrrolo­acridines are a type of fused heterocyclic compounds that combine the structures of pyrrole and acridine. As a result of their appealing biological and therapeutic properties attributed to their ability to inter­calate DNA (Belmont *et al.*, 2007 ▸), organic chemists are currently paying close attention to the synthesis of these compounds through multi-step procedures (Dandia *et al.* 2015 ▸; Hao *et al.* 2013 ▸; Jiang *et al.* 2012 ▸; Ray *et al.* 2014 ▸; Wang *et al.* 2012 ▸). As part of our work in this area, we now describe the synthesis and structure of the title compound, C_22_H_18_N_2_O.

**Figure 1:**
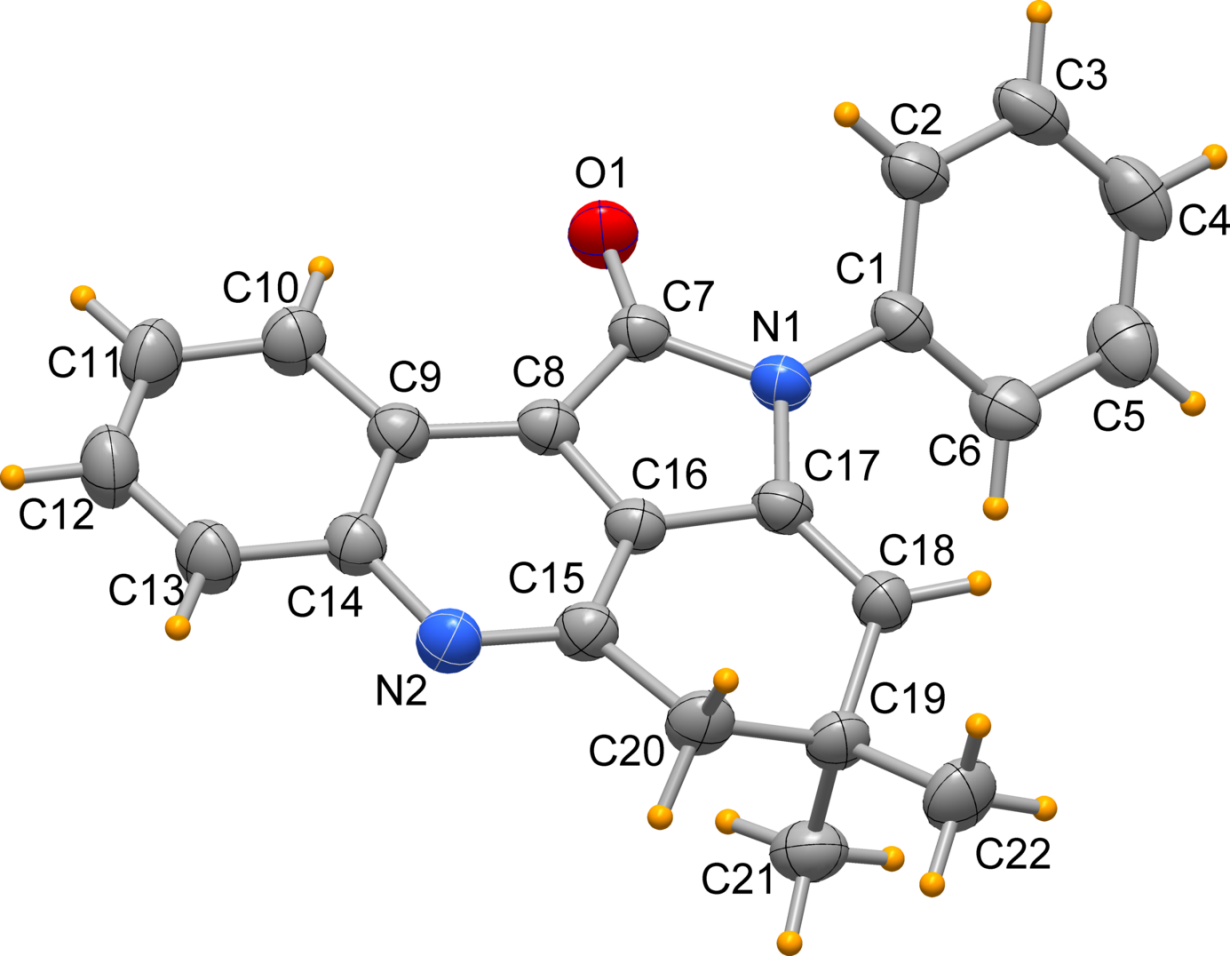
The mol­ecular structure of the title mol­ecule (displacement ellipsoids at the 50% probability level).

The mol­ecular structure of the title mol­ecule is shown in Fig. 1 ▸. The chemical structure consists of a central acridine core fused to a pyrrolidone ring, a combination of rings that contributes to its planarity with the exception of the *sp*^3^ carbon atoms (C19, C20 displaced by 0.445 (2), and −0.157 (2) Å, respectively), and of the C1–C6 phenyl ring bound to the pyrrol N atom. The carbonyl C=O bond length is 1.2187 (15) Å, and all other bond distances are as expected. The phenyl ring forms a dihedral angle of 43.85 (1)° with the mean plane through the acridin moiety. This conformation is similar to that found in the structures having a 3-nitro­phenyl or 4-methyl­phenyl ring replacing the phenyl ring, where the corresponding dihedral angles are 48.84 (5) and 44.72 (4)°, respectively (Hao *et al.* 2013 ▸). The 9-fluoro derivative (Dandia *et al.* 2015 ▸) has the phenyl ring tilted by 49.32 (1)° with respect to the acridin fragment. Thus all the cited analogous derivatives exhibit similar conformations.

**Figure 2:**
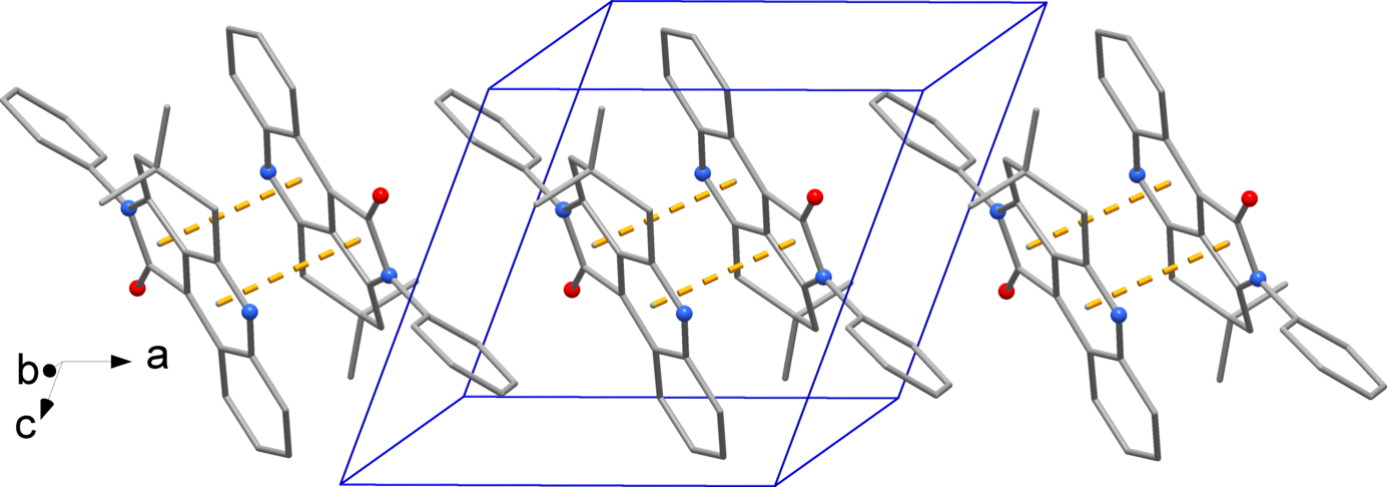
Detail of the crystal packing showing the π-stacking inter­actions as dashed lines (H atoms not indicated for the sake of clarity).

The crystal packing evidences π–π-stacked dimers with a centroid-to-centroid distance of 3.489 (2) Å (Fig. 2 ▸). In addition, a weak C3—H3⋯O1(2 − *x*, 2 − *y*, 1 − *z*) hydrogen bond is observed with H⋯O = 2.53 Å, C⋯O = 3.438 (2) Å and C—H⋯O = 165°.

## Synthesis and crystallization

Dimedone (1.00 mmol), aniline (1.00 mmol) and nicotinic acid (5–10 mol %) were mixed in 5.0 ml of toluene and the reaction mixture was heated over an oil-bath at reflux conditions with an efficient CaCl_2_ guard-tube for 6–8 h. Isatin (1.00 mmol) was added sequentially to the reaction mixture and it was heated to reflux till the completion of the reaction that was monitored by TLC with UV detector at 365 nm. Then, the solvent was evaporated and the residue purified by column chromatography (acetone/petroleum ether, 1:6). Single crystals suitable for X-ray analysis were obtained from slow evaporation of an ethano­lic solution of the product containing few drops of acetone: colour: light brown, yield: 80%, melting point: 191–193°C

^1^H NMR (400 MHz, CDCl_3_): *δ*_H_ (p.p.m.)= 8.73 (*dd*, *J* = 8, 8 Hz, 1H, Ar—H), 8.16 (*d*, *J* = 8 Hz, 1H, Ar—H), 7.83 (*m*, 1H, Ar—H), 7.75 (*m*, 1H, Ar—H), 7.68–7.54 (*m*, 4H, Ar—H), 7.53–7.41 (1*H*, Ar—H), 5.63 (*s*, 1H, ali-H), 3.22 (*s*, 2H, ali-H), 1.33 (*s*, 6H, ali-H).

^13^C NMR (100 MHz, CDCl_3_): *δ*_C_ (p.p.m.), 166.75, 154.60, 149.73, 134.78, 133.37, 129.55, 129.46, 129.39, 127.80, 127.48, 126.46, 126.40, 124.99, 124.25, 122.63, 118.36, 44.21, 37.11, 30.89.

HRMS(ESI): m/*z* [*M* + H]^+^ calculated for C_22_H_19_N_2_O: 327.399, found 327.1491.

## Refinement

Crystal data, data collection and structure refinement are summarized in Table 1 ▸.

## Supplementary Material

Crystal structure: contains datablock(s) I. DOI: 10.1107/S241431462500361X/hb4513sup1.cif

Structure factors: contains datablock(s) I. DOI: 10.1107/S241431462500361X/hb4513Isup2.hkl

Supporting information file. DOI: 10.1107/S241431462500361X/hb4513Isup3.cml

CCDC reference: 2442640

Additional supporting information:  crystallographic information; 3D view; checkCIF report

## Figures and Tables

**Table 1:** Experimental details

Crystal data	
Chemical formula	C_22_H_18_N_2_O
*M*_r_	326.38
Crystal system, space group	Triclinic, *P* 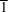
Temperature (K)	297
*a*, *b*, *c* (Å)	9.430 (3), 9.997 (4), 10.203 (4)
α, β, γ (°)	99.833 (14), 107.559 (14), 106.866 (14)
*V* (Å^3^)	841.8 (6)
*Z*	2
Radiation type	Mo *K*α
μ (mm^−1^)	0.08
Crystal size (mm)	0.30 × 0.30 × 0.28

Data collection	
Diffractometer	Bruker APEXII CCD
No. of measured, independent and observed [*I* > 2σ(*I*)] reflections	22780, 3806, 3047
*R*_int_	0.041
(sin θ/λ)_max_ (Å^−1^)	0.650

Refinement	
*R*[*F*^2^ > 2σ(*F*^2^)], *wR*(*F*^2^), *S*	0.053, 0.138, 1.12
No. of reflections	3806
No. of parameters	228
H-atom treatment	H-atom parameters constrained
Δρ_max_, Δρ_min_ (e Å^−3^)	0.37, −0.49

Computer programs: *APEX2* and *SAINT* (Bruker, 2012 ▸), *SHELXT2014/5* (Sheldrick, 2015*a* ▸), *SHELXL2019/2* (Sheldrick, 2015*b* ▸) and *DIAMOND* (Brandenburg & Putz, 1999 ▸).

## full crystallographic data

### 11,11-Dimethyl-14-phenyl-8,14-diazatetracyclo[7.6.1.02,7.013,16]hexadeca-1(16),2(7),3,5,8,12-hexaen-15-one Crystal data

**Table:**

C_22_H_18_N_2_O	*Z* = 2
*M**_r_* = 326.38	*F*(000) = 344
Triclinic, *P*1	*D*_x_ = 1.288 Mg m^−^^3^
*a* = 9.430 (3) Å	Mo *K*α radiation, λ = 0.71073 Å
*b* = 9.997 (4) Å	Cell parameters from 9587 reflections
*c* = 10.203 (4) Å	θ = 2.2–26.7°
α = 99.833 (14)°	µ = 0.08 mm^−^^1^
β = 107.559 (14)°	*T* = 297 K
γ = 106.866 (14)°	Block, brown
*V* = 841.8 (6) Å^3^	0.30 × 0.30 × 0.28 mm

### 11,11-Dimethyl-14-phenyl-8,14-diazatetracyclo[7.6.1.02,7.013,16]hexadeca-1(16),2(7),3,5,8,12-hexaen-15-one Data collection

**Table:**

Bruker APEXII CCD diffractometer	*R*_int_ = 0.041
Radiation source: sealed tube	θ_max_ = 27.5°, θ_min_ = 2.2°
φ and ω scans	*h* = −12→12
22780 measured reflections	*k* = −12→12
3806 independent reflections	*l* = −13→13
3047 reflections with *I* > 2σ(*I*)	

### 11,11-Dimethyl-14-phenyl-8,14-diazatetracyclo[7.6.1.02,7.013,16]hexadeca-1(16),2(7),3,5,8,12-hexaen-15-one Refinement

**Table:**

Refinement on *F*^2^	Primary atom site location: dual
Least-squares matrix: full	Hydrogen site location: inferred from neighbouring sites
*R*[*F*^2^ > 2σ(*F*^2^)] = 0.053	H-atom parameters constrained
*wR*(*F*^2^) = 0.138	*w* = 1/[σ^2^(*F*_o_^2^) + (0.0778*P*)^2^ + 0.0906*P*] where *P* = (*F*_o_^2^ + 2*F*_c_^2^)/3
*S* = 1.12	(Δ/σ)_max_ < 0.001
3806 reflections	Δρ_max_ = 0.37 e Å^−^^3^
228 parameters	Δρ_min_ = −0.48 e Å^−^^3^
0 restraints	

### 11,11-Dimethyl-14-phenyl-8,14-diazatetracyclo[7.6.1.02,7.013,16]hexadeca-1(16),2(7),3,5,8,12-hexaen-15-one Special details

**Table:**

Geometry. All esds (except the esd in the dihedral angle between two l.s. planes) are estimated using the full covariance matrix. The cell esds are taken into account individually in the estimation of esds in distances, angles and torsion angles; correlations between esds in cell parameters are only used when they are defined by crystal symmetry. An approximate (isotropic) treatment of cell esds is used for estimating esds involving l.s. planes.
Refinement. H atoms were located at geometrical positions and refined as riding atoms.

### 11,11-Dimethyl-14-phenyl-8,14-diazatetracyclo[7.6.1.02,7.013,16]hexadeca-1(16),2(7),3,5,8,12-hexaen-15-one Fractional atomic coordinates and isotropic or equivalent isotropic displacement parameters (Å^2^)

**Table:**

	*x*	*y*	*z*	*U*_iso_*/*U*_eq_	
O1	0.77493 (12)	0.67107 (10)	0.34393 (10)	0.0482 (3)	
N1	0.85067 (12)	0.60933 (10)	0.55938 (10)	0.0351 (3)	
N2	0.39760 (12)	0.19595 (11)	0.39073 (12)	0.0404 (3)	
C1	0.99874 (14)	0.72885 (12)	0.63425 (12)	0.0346 (3)	
C2	1.00582 (16)	0.86935 (13)	0.62965 (14)	0.0409 (3)	
H2	0.914571	0.884859	0.579572	0.049*	
C3	1.14935 (18)	0.98514 (14)	0.70003 (15)	0.0502 (4)	
H3	1.155281	1.078553	0.695182	0.060*	
C4	1.28445 (18)	0.96283 (16)	0.77777 (17)	0.0560 (4)	
H4	1.380240	1.041467	0.826627	0.067*	
C5	1.27703 (17)	0.82358 (17)	0.78282 (17)	0.0538 (4)	
H5	1.367944	0.809007	0.835261	0.065*	
C6	1.13471 (16)	0.70562 (14)	0.71007 (15)	0.0442 (3)	
H6	1.130304	0.611866	0.712011	0.053*	
C7	0.74945 (15)	0.59113 (12)	0.41867 (13)	0.0347 (3)	
C8	0.61124 (14)	0.45417 (12)	0.38158 (12)	0.0328 (3)	
C9	0.46972 (14)	0.37668 (13)	0.25861 (12)	0.0344 (3)	
C10	0.42702 (17)	0.41674 (15)	0.12990 (14)	0.0429 (3)	
H10	0.493807	0.501397	0.121915	0.052*	
C11	0.28797 (18)	0.33172 (17)	0.01693 (14)	0.0515 (4)	
H11	0.261021	0.358521	−0.067512	0.062*	
C12	0.18646 (18)	0.20460 (17)	0.02846 (15)	0.0526 (4)	
H12	0.092101	0.147652	−0.048569	0.063*	
C13	0.22420 (16)	0.16301 (15)	0.15150 (15)	0.0475 (3)	
H13	0.154790	0.078417	0.157119	0.057*	
C14	0.36693 (15)	0.24652 (13)	0.27015 (13)	0.0368 (3)	
C15	0.53139 (14)	0.27159 (13)	0.50142 (13)	0.0357 (3)	
C16	0.63918 (14)	0.39923 (12)	0.49598 (12)	0.0325 (3)	
C17	0.78949 (14)	0.48942 (12)	0.60979 (12)	0.0326 (3)	
C18	0.84327 (16)	0.44642 (13)	0.72607 (13)	0.0387 (3)	
H18	0.933264	0.509730	0.804289	0.046*	
C19	0.75654 (16)	0.29286 (13)	0.73030 (13)	0.0389 (3)	
C20	0.57421 (16)	0.23479 (15)	0.64175 (14)	0.0420 (3)	
H20A	0.532291	0.129698	0.622742	0.050*	
H20B	0.522641	0.274877	0.698854	0.050*	
C21	0.83069 (18)	0.19303 (15)	0.66618 (16)	0.0499 (4)	
H21A	0.786913	0.097045	0.675034	0.075*	
H21B	0.944299	0.231561	0.716766	0.075*	
H21C	0.807088	0.188238	0.566775	0.075*	
C22	0.7832 (2)	0.29030 (18)	0.88549 (15)	0.0551 (4)	
H22A	0.737958	0.353005	0.926232	0.083*	
H22B	0.895682	0.323682	0.940287	0.083*	
H22C	0.732800	0.192598	0.887174	0.083*	

### 11,11-Dimethyl-14-phenyl-8,14-diazatetracyclo[7.6.1.02,7.013,16]hexadeca-1(16),2(7),3,5,8,12-hexaen-15-one Atomic displacement parameters (Å^2^)

**Table:**

	*U*^11^	*U*^22^	*U*^33^	*U*^12^	*U*^13^	*U*^23^
O1	0.0543 (6)	0.0394 (5)	0.0430 (5)	0.0031 (4)	0.0157 (4)	0.0210 (4)
N1	0.0367 (6)	0.0278 (5)	0.0364 (5)	0.0061 (4)	0.0118 (4)	0.0115 (4)
N2	0.0362 (6)	0.0375 (6)	0.0434 (6)	0.0065 (5)	0.0132 (5)	0.0163 (5)
C1	0.0364 (7)	0.0288 (6)	0.0358 (6)	0.0063 (5)	0.0163 (5)	0.0064 (5)
C2	0.0475 (7)	0.0315 (6)	0.0423 (7)	0.0109 (5)	0.0183 (6)	0.0105 (5)
C3	0.0618 (9)	0.0289 (6)	0.0526 (8)	0.0053 (6)	0.0244 (7)	0.0073 (6)
C4	0.0456 (8)	0.0440 (8)	0.0585 (9)	−0.0038 (6)	0.0194 (7)	0.0011 (7)
C5	0.0364 (7)	0.0560 (9)	0.0589 (9)	0.0108 (6)	0.0147 (6)	0.0080 (7)
C6	0.0412 (7)	0.0369 (7)	0.0532 (8)	0.0133 (6)	0.0184 (6)	0.0099 (6)
C7	0.0382 (6)	0.0302 (6)	0.0351 (6)	0.0100 (5)	0.0138 (5)	0.0111 (5)
C8	0.0351 (6)	0.0305 (6)	0.0350 (6)	0.0108 (5)	0.0155 (5)	0.0120 (5)
C9	0.0352 (6)	0.0335 (6)	0.0347 (6)	0.0116 (5)	0.0141 (5)	0.0100 (5)
C10	0.0452 (7)	0.0439 (7)	0.0367 (6)	0.0101 (6)	0.0148 (6)	0.0153 (5)
C11	0.0532 (9)	0.0582 (9)	0.0341 (7)	0.0136 (7)	0.0096 (6)	0.0150 (6)
C12	0.0437 (8)	0.0550 (8)	0.0388 (7)	0.0057 (6)	0.0038 (6)	0.0062 (6)
C13	0.0395 (7)	0.0429 (7)	0.0461 (7)	0.0023 (6)	0.0104 (6)	0.0106 (6)
C14	0.0338 (6)	0.0351 (6)	0.0380 (6)	0.0085 (5)	0.0129 (5)	0.0104 (5)
C15	0.0331 (6)	0.0339 (6)	0.0405 (6)	0.0093 (5)	0.0145 (5)	0.0152 (5)
C16	0.0336 (6)	0.0295 (6)	0.0348 (6)	0.0098 (5)	0.0136 (5)	0.0113 (5)
C17	0.0359 (6)	0.0267 (5)	0.0350 (6)	0.0094 (5)	0.0143 (5)	0.0098 (5)
C18	0.0401 (7)	0.0334 (6)	0.0348 (6)	0.0078 (5)	0.0088 (5)	0.0096 (5)
C19	0.0434 (7)	0.0366 (6)	0.0366 (6)	0.0115 (5)	0.0132 (5)	0.0184 (5)
C20	0.0414 (7)	0.0405 (7)	0.0438 (7)	0.0084 (6)	0.0165 (6)	0.0216 (6)
C21	0.0546 (8)	0.0415 (7)	0.0583 (8)	0.0198 (6)	0.0212 (7)	0.0214 (6)
C22	0.0647 (10)	0.0584 (9)	0.0421 (7)	0.0191 (8)	0.0166 (7)	0.0259 (7)

### 11,11-Dimethyl-14-phenyl-8,14-diazatetracyclo[7.6.1.02,7.013,16]hexadeca-1(16),2(7),3,5,8,12-hexaen-15-one Geometric parameters (Å, º)

**Table:**

O1—C7	1.2187 (15)	C11—C12	1.399 (2)
N1—C7	1.4102 (17)	C11—H11	0.9300
N1—C17	1.4222 (15)	C12—C13	1.368 (2)
N1—C1	1.4275 (16)	C12—H12	0.9300
N2—C15	1.3169 (17)	C13—C14	1.4120 (18)
N2—C14	1.3912 (17)	C13—H13	0.9300
C1—C6	1.3900 (19)	C15—C16	1.4037 (17)
C1—C2	1.3962 (18)	C15—C20	1.5052 (18)
C2—C3	1.381 (2)	C16—C17	1.4416 (17)
C2—H2	0.9300	C17—C18	1.3369 (17)
C3—C4	1.385 (2)	C18—C19	1.5307 (18)
C3—H3	0.9300	C18—H18	0.9300
C4—C5	1.384 (2)	C19—C22	1.5325 (19)
C4—H4	0.9300	C19—C21	1.540 (2)
C5—C6	1.387 (2)	C19—C20	1.5538 (19)
C5—H5	0.9300	C20—H20A	0.9700
C6—H6	0.9300	C20—H20B	0.9700
C7—C8	1.4855 (17)	C21—H21A	0.9600
C8—C16	1.3605 (17)	C21—H21B	0.9600
C8—C9	1.4189 (17)	C21—H21C	0.9600
C9—C10	1.4147 (18)	C22—H22A	0.9600
C9—C14	1.4269 (18)	C22—H22B	0.9600
C10—C11	1.370 (2)	C22—H22C	0.9600
C10—H10	0.9300		
			
C7—N1—C17	110.79 (10)	C12—C13—H13	119.5
C7—N1—C1	123.16 (10)	C14—C13—H13	119.5
C17—N1—C1	125.95 (10)	N2—C14—C13	117.52 (11)
C15—N2—C14	118.19 (11)	N2—C14—C9	124.43 (11)
C6—C1—C2	120.32 (12)	C13—C14—C9	118.04 (12)
C6—C1—N1	120.42 (11)	N2—C15—C16	120.22 (11)
C2—C1—N1	119.26 (11)	N2—C15—C20	123.64 (11)
C3—C2—C1	119.52 (13)	C16—C15—C20	116.03 (11)
C3—C2—H2	120.2	C8—C16—C15	123.23 (12)
C1—C2—H2	120.2	C8—C16—C17	111.80 (11)
C2—C3—C4	120.34 (13)	C15—C16—C17	124.96 (11)
C2—C3—H3	119.8	C18—C17—N1	135.06 (11)
C4—C3—H3	119.8	C18—C17—C16	120.24 (11)
C5—C4—C3	120.05 (13)	N1—C17—C16	104.60 (10)
C5—C4—H4	120.0	C17—C18—C19	119.91 (11)
C3—C4—H4	120.0	C17—C18—H18	120.0
C4—C5—C6	120.36 (14)	C19—C18—H18	120.0
C4—C5—H5	119.8	C18—C19—C22	110.21 (11)
C6—C5—H5	119.8	C18—C19—C21	106.57 (11)
C5—C6—C1	119.39 (13)	C22—C19—C21	109.13 (11)
C5—C6—H6	120.3	C18—C19—C20	112.81 (10)
C1—C6—H6	120.3	C22—C19—C20	109.02 (11)
O1—C7—N1	126.03 (11)	C21—C19—C20	109.02 (11)
O1—C7—C8	128.23 (12)	C15—C20—C19	114.02 (10)
N1—C7—C8	105.72 (10)	C15—C20—H20A	108.7
C16—C8—C9	119.06 (11)	C19—C20—H20A	108.7
C16—C8—C7	106.99 (11)	C15—C20—H20B	108.7
C9—C8—C7	133.94 (11)	C19—C20—H20B	108.7
C10—C9—C8	125.67 (12)	H20A—C20—H20B	107.6
C10—C9—C14	119.51 (12)	C19—C21—H21A	109.5
C8—C9—C14	114.81 (11)	C19—C21—H21B	109.5
C11—C10—C9	120.48 (13)	H21A—C21—H21B	109.5
C11—C10—H10	119.8	C19—C21—H21C	109.5
C9—C10—H10	119.8	H21A—C21—H21C	109.5
C10—C11—C12	120.07 (13)	H21B—C21—H21C	109.5
C10—C11—H11	120.0	C19—C22—H22A	109.5
C12—C11—H11	120.0	C19—C22—H22B	109.5
C13—C12—C11	120.87 (13)	H22A—C22—H22B	109.5
C13—C12—H12	119.6	C19—C22—H22C	109.5
C11—C12—H12	119.6	H22A—C22—H22C	109.5
C12—C13—C14	121.01 (13)	H22B—C22—H22C	109.5
			
C7—N1—C1—C6	−135.02 (13)	C10—C9—C14—N2	179.36 (11)
C17—N1—C1—C6	40.75 (17)	C8—C9—C14—N2	0.47 (18)
C7—N1—C1—C2	44.53 (16)	C10—C9—C14—C13	−0.68 (18)
C17—N1—C1—C2	−139.71 (12)	C8—C9—C14—C13	−179.57 (11)
C6—C1—C2—C3	0.56 (19)	C14—N2—C15—C16	−0.26 (18)
N1—C1—C2—C3	−178.99 (11)	C14—N2—C15—C20	175.80 (11)
C1—C2—C3—C4	−1.7 (2)	C9—C8—C16—C15	−2.69 (18)
C2—C3—C4—C5	1.4 (2)	C7—C8—C16—C15	178.19 (11)
C3—C4—C5—C6	0.1 (2)	C9—C8—C16—C17	178.58 (10)
C4—C5—C6—C1	−1.2 (2)	C7—C8—C16—C17	−0.54 (13)
C2—C1—C6—C5	0.9 (2)	N2—C15—C16—C8	2.16 (19)
N1—C1—C6—C5	−179.56 (11)	C20—C15—C16—C8	−174.19 (11)
C17—N1—C7—O1	−175.27 (12)	N2—C15—C16—C17	−179.28 (11)
C1—N1—C7—O1	1.07 (19)	C20—C15—C16—C17	4.37 (18)
C17—N1—C7—C8	3.10 (13)	C7—N1—C17—C18	172.77 (13)
C1—N1—C7—C8	179.43 (10)	C1—N1—C17—C18	−3.4 (2)
O1—C7—C8—C16	176.78 (12)	C7—N1—C17—C16	−3.37 (13)
N1—C7—C8—C16	−1.54 (13)	C1—N1—C17—C16	−179.58 (10)
O1—C7—C8—C9	−2.2 (2)	C8—C16—C17—C18	−174.48 (11)
N1—C7—C8—C9	179.53 (12)	C15—C16—C17—C18	6.82 (19)
C16—C8—C9—C10	−177.48 (11)	C8—C16—C17—N1	2.37 (13)
C7—C8—C9—C10	1.4 (2)	C15—C16—C17—N1	−176.33 (11)
C16—C8—C9—C14	1.34 (17)	N1—C17—C18—C19	−167.75 (12)
C7—C8—C9—C14	−179.83 (12)	C16—C17—C18—C19	7.93 (18)
C8—C9—C10—C11	178.88 (12)	C17—C18—C19—C22	−153.29 (12)
C14—C9—C10—C11	0.1 (2)	C17—C18—C19—C21	88.42 (15)
C9—C10—C11—C12	0.3 (2)	C17—C18—C19—C20	−31.17 (17)
C10—C11—C12—C13	−0.2 (2)	N2—C15—C20—C19	155.80 (12)
C11—C12—C13—C14	−0.4 (2)	C16—C15—C20—C19	−27.98 (16)
C15—N2—C14—C13	179.03 (12)	C18—C19—C20—C15	40.35 (16)
C15—N2—C14—C9	−1.02 (19)	C22—C19—C20—C15	163.13 (11)
C12—C13—C14—N2	−179.24 (12)	C21—C19—C20—C15	−77.82 (14)
C12—C13—C14—C9	0.8 (2)		

## References

[bb1] Belmont, P., Bosson, J., Godet, T. & Tiano, M. (2007). *Anticancer Agents Med. Chem.* **7**, 139–169. 10.2174/187152007780058669 17348825

[bb2] Brandenburg, K. & Putz, H. (1999). *DIAMOND*. Crystal Impact GbR, Bonn, Germany.

[bb3] Bruker (2012). *APEX2* and *SAINT*. Bruker AXS Inc., Madison, Wisconsin, USA.

[bb4] Dandia, A., Sharma, A., Parewa, V., Kumawat, B., Rathore, K. S. & Sharma, A. (2015). *RSC Adv.* **5**, 91888–91902.

[bb5] Hao, W.-J., Wang, J.-Q., Xu, X.-P., Zhang, S.-L., Wang, S.-Y. & Ji, S.-J. (2013). *J. Org. Chem.* **78**, 12362–12373. 10.1021/jo401773j 24295532

[bb6] Jiang, B., Wang, X., Li, M.-Y., Wu, Q., Ye, Q., Xu, H.-W. & Tu, S.-J. (2012). *Org. Biomol. Chem.* **10**, 8533–8538. 10.1039/c2ob26315g 23011183

[bb7] Ray, S., Bhaumik, A., Pramanik, M. & Mukhopadhyay, C. (2014). *RSC Adv.* **4**, 15441–15450.

[bb8] Sheldrick, G. M. (2015*a*). *Acta Cryst.* A**71**, 3–8.

[bb9] Sheldrick, G. M. (2015*b*). *Acta Cryst.* C**71**, 3–8.

[bb10] Wang, H., Li, L., Lin, W., Xu, P., Huang, Z. & Shi, D. (2012). *Org. Lett.* **14**, 4598–4601. 10.1021/ol302058g 22920713

